# Supplementary material for: Splice-Junction-Based Mapping of Alternative Isoforms in the Human Proteome
Source: Cell Rep. Author manuscript; Available in PMC 2020 Jan 15. (PMC6961840; doi:10.1016/j.celrep.2019.11.026)
Supplement: 2 [file NIHMS1546469-supplement-2.zip › DF1/PXD010154/Esophagus/Esophagus_36_LUC7L3_ENTDLEVGIEEDQKAG.pdf]

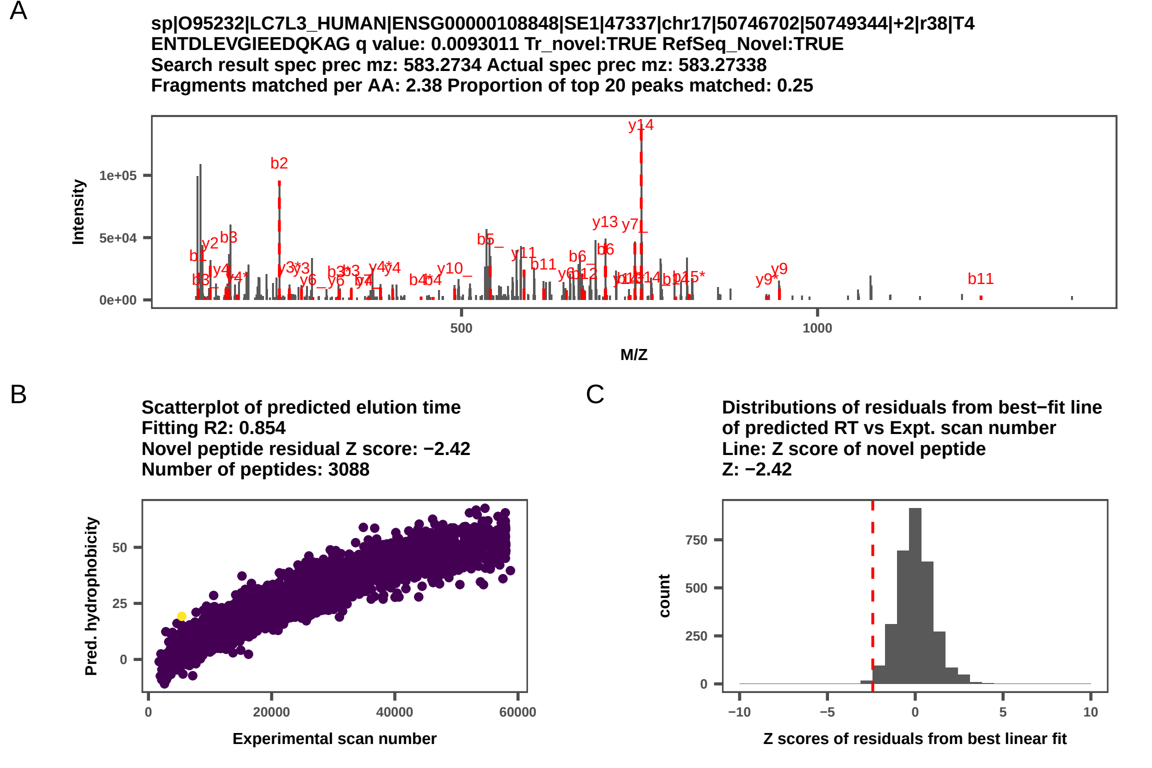
A
sp|O95232|LC7L3_HUMAN|ENSG00000108848|SE1|47337|chr17|50746702|50749344|+2|r38|T4
ENTDLEVGIEEDQKAG q value: 0.0093011 Tr_novel:TRUE RefSeq_Novel:TRUE
Search result spec prec mz: 583.2734 Actual spec prec mz: 583.27338
Fragments matched per AA: 2.38 Proportion of top 20 peaks matched: 0.25
Intensity
1e+05
5e+04
0e+00
M/Z
500
1000
y14
b2
y13
y7
b5
y11
b11
b6
y9
y9*
b11
B
Scatterplot of predicted elution time
Fitting R2: 0.854
Novel peptide residual Z score: −2.42
Number of peptides: 3088
Pred. hydrophobicity
50
25
0
0
20000
40000
60000
Experimental scan number
C
Distributions of residuals from best−fit line
of predicted RT vs Expt. scan number
Line: Z score of novel peptide
Z: −2.42
count
750
500
250
0
−10
−5
0
5
10
Z scores of residuals from best linear fit
